# Late surgical repair of a traumatic ventricular septal defect

**DOI:** 10.1186/s13019-014-0145-1

**Published:** 2014-09-20

**Authors:** Leanne Harling, Hutan Ashrafian, Roberto P Casula, Thanos Athanasiou

**Affiliations:** Department of Cardiothoracic Surgery, Imperial College Healthcare NHS Trust, Hammersmith Hospital, Du Cane Road, London, W12 0HS UK; Department of Surgery and Cancer, Imperial College London, 10th Floor QEQM Building, St Mary’s Hospital, Praed Street, London, W2 1NY UK

**Keywords:** Ventricular septal defect, Trauma, Shunt, Surgery

## Abstract

Ventricular Septal Defect (VSD) complicates approximately 1-5% of patients presenting with penetrating chest trauma, however not all VSDs are evident at the time of initial presentation to the emergency department. Acute closure of traumatic VSDs is indicated in patients with a large defect and haemodynamic compromise, however closure may be delayed in smaller defects in order to minimise operative time, reduce operative mortality and allow for recovery from the initial trauma. We describe the case of a previously healthy 23 year-old Caucasian man who presented *in extremis* following stab wounds to the precordium. After emergency cardiopulmonary bypass and closure of lacerations to both the left and right ventricles, postoperative trans-thoracic echocardiography (TTE) noted a restrictive intramuscular VSD with a high velocity left to right shunt, initially managed conservatively. Elective surgical closure was performed 10 months after the initial injury through a right ventriculotomy using 4–0 Proline sutures reinforced with Teflon pledgets. Despite excellent clinical recovery, 3-month follow-up TTE noted a residual VSD in the mid apical septum. However, given the presence of minimal left to right shunt and the small size of the defect, the patient was managed conservatively with annual review and repeat transthoracic echo. This case highlights the potential pitfalls in both the diagnosis and management of traumatic VSDs particularly where the patient presents *in extremis* with other life-threatening injuries. Furthermore, it exemplifies the importance of a multidisciplinary approach when planning any elective intervention. Regardless of the management strategy, repeated re-assessment and re-evaluation is vital following penetrating cardiac trauma, and vigilant long-term follow-up is of paramount importance in these cases.

**Electronic supplementary material:**

The online version of this article (doi:10.1186/s13019-014-0145-1) contains supplementary material, which is available to authorized users.

## Background

Chest trauma accounts for approximately 12% of all penetrating knife injuries in the United Kingdom [1]. Although the right ventricular free wall is the most common site of myocardial injury, approximately 1-5% of patients present with traumatic ventricular septal defects (VSD) [2]. VSD may occur not only as a result of direct septal laceration, but also secondary to blunt trauma [3], deceleration injury [4], and septal ischaemia with subsequent necrosis sustained as a result of either direct coronary artery injury or acute thrombosis [5]. The primary management in these patients is resuscitation and repair of any penetrating injury, which may be performed either with or without cardiopulmonary bypass. Acute closure of traumatic VSDs may be indicated in patients with a large defect and haemodynamic compromise, however closure is often delayed in the case of smaller defects in order to minimise operative time, reduce operative mortality and allow for recovery from the initial trauma [6]. Furthermore, spontaneous closure of small traumatic VSDs has been reported, and thus defects with a pulmonary to systemic ratio below 1.5:1 may be managed conservatively [7],[8]. Definitive closure by means of either a percutaneous device or open patch repair remains the mainstay of management in large or symptomatic VSDs.

We present the case of a young, otherwise healthy, Caucasian male presenting with a moderate to large muscular VSD secondary to penetrating chest trauma which subsequently underwent direct surgical closure. This case highlights a number of potential pitfalls in the diagnosis and management of traumatic VSDs and demonstrates the requirement for careful multi-modality imaging in both the early diagnosis and follow-up of these patients.

## Case presentation

A previously healthy 23 year-old Caucasian man was brought to the emergency department of our acute trauma centre following stab wounds to the precordium. He arrived *in extremis,* in cardiac arrest with profound metabolic acidosis (pH 7.2 and Lactate 12) and cardiac tamponade, and was taken immediately to the operating theatre. Emergency sternotomy was performed and after initiating cardiopulmonary bypass via the ascending aorta and right atrium, penetrating wounds to both the left and right ventricles were repaired. Although the anterior and posterior ventricular lacerations were in close proximity to left anterior descending (LAD) and obtuse marginal (OM) coronary arteries respectively, no direct arterial injury was identified and the patient was weaned from cardiopulmonary bypass without difficulty. Intra-operative transoesophageal echo (TOE) was performed demonstrating apical akinesis secondary to the penetrating injury, although only limited views were achieved. Post-operative transthoracic echocardiography (TTE) later showed a restrictive Ventricular Septal Defect (VSD) in the muscular intraventricular septum with a high velocity left to right shunt. There was mildly impaired LV systolic function and moderate to severely impaired RV systolic function. Given the patient’s condition it was decided to manage the VSD conservatively and plan a late stage repair as required. On post-operative day 4, the patient suffered a large embolic right middle cerebral artery (MCA) infarction resulting in malignant MCA syndrome and requiring emergency decompressive craniotomy. Despite subsequent left sided weakness and inattention, he recovered well and fibre-titanium plate repair of the craniotomy was undertaken after 4 months. Eight months following the initial trauma the patient returned for re-assessment of the VSD. Clinical assessment revealed mild breathlessness (NYHA II) although no murmur or signs of cardiac failure were evident. The patient’s mobility remained limited secondary to on-going left sided weakness. Trans-oesophageal echo demonstrated a moderate sized unrestrictive VSD (12 × 18 mm; PVel 2.5 m/s; PGrad 25 mmHg) in the apical muscular interventricular septum. There was a left to right shunt with pulmonary to aortic flow of 2:1. The right ventricle appeared mildly dilated, with good overall systolic function. The left ventricular dimensions had increased significantly since the initial post-operative TTE with the diastolic LV internal dimension increasing from 4.1 to 5.2 cm and the systolic LV internal dimension increasing from 2.9 to 3.7 cm. The LV systolic function was normal. Cardiac MRI also demonstrated a left to right shunt with a systemic to pulmonary flow (Qp:Qs) of 1.8:1. Focal filling with transmural scarring on the apical RV free wall and mid infero-lateral wall was evident in keeping with the knife entry/exit and patch repair. Multidisciplinary assessment concluded that although device closure would have been possible, it would have been technically challenging due to the presence of a prominent adjacent moderator band and trabecular tissue.

### Surgical procedure

**Figure 1:**
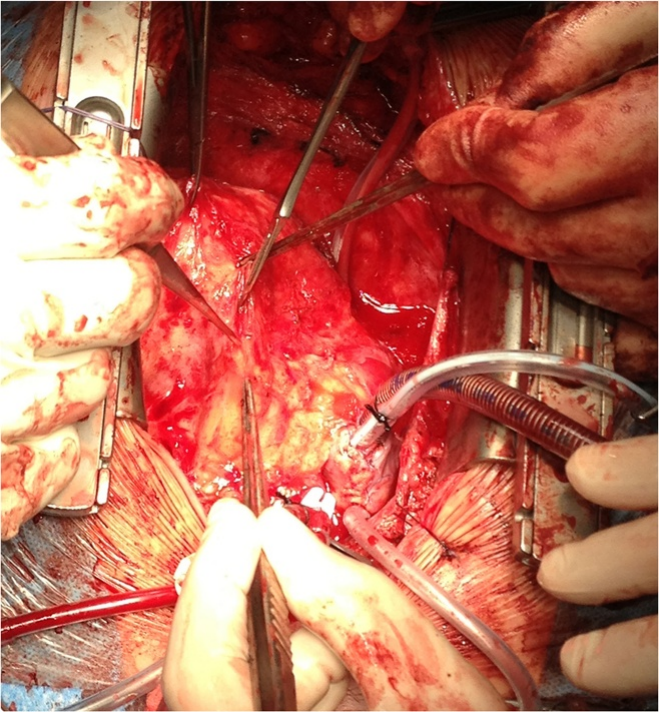
**Surgical approach to the VSD via right ventriculotomy.**

**Figure 2:**
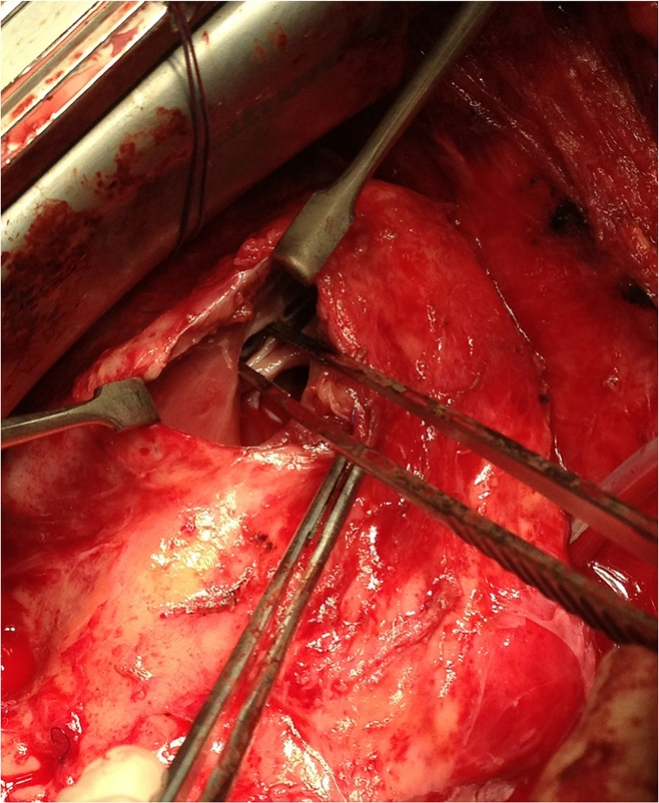
**Direct intraoperative visualisation of the VSD.**

**Figure 3:**
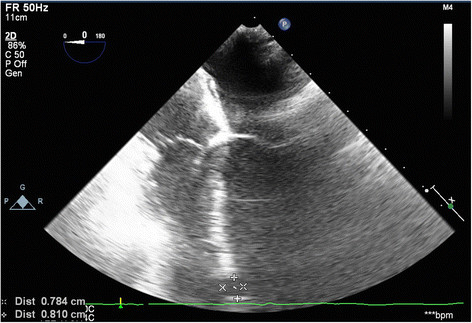
**Intraoperative 0 degree 4 chamber TOE view confirming the position of a large VSD in the apical muscular interventricular septum.**

**Figure 4:**
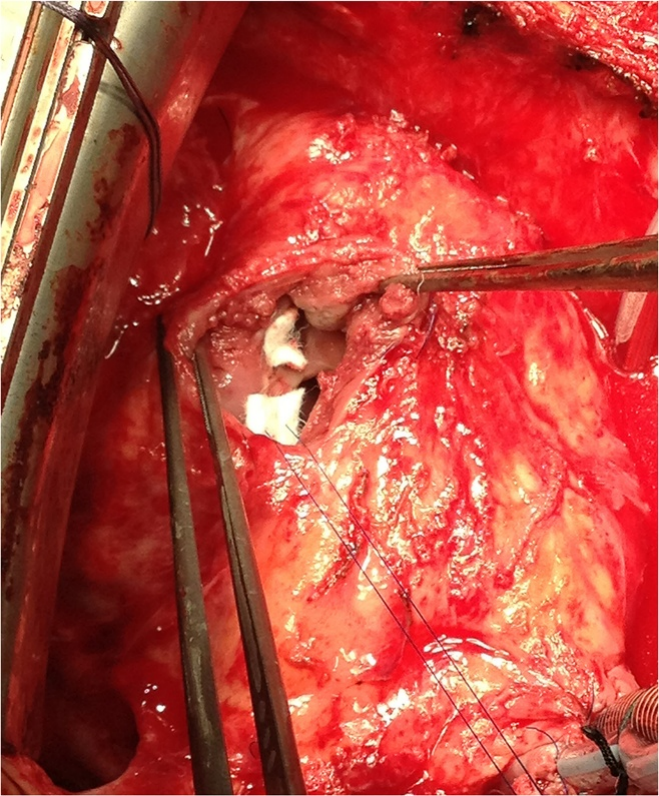
**Direct closure of the VSD using pledgeted sutures.**

**Figure 5:**
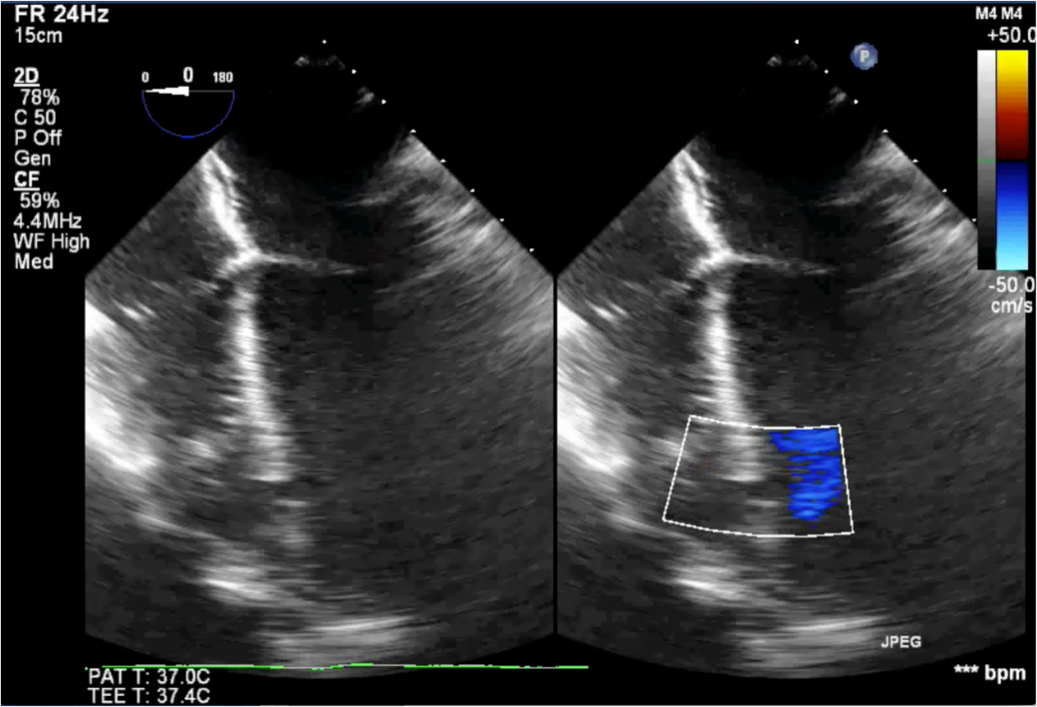
**Intra-operative TOE images demonstrating absence of colour doppler flow across region of VSD after direct surgical closure of the defect.**

Surgical closure of the VSD was performed via redo median sternotomy 10 months after the initial injury. Venous cannulation was via the left femoral vein and right atrium and arterial cannulation was directly via the aorta. Due to the apical location of the VSD in the muscular septum, the approach was via a right ventriculotomy (Figure 1). This enabled direct visualisation of the VSD (Figure 2), which was confirmed by intraoperative TOE (Figure 3). The defect was closed under direct vision using 4–0 Prolene sutures reinforced with Teflon pledgets (Figure 4). The ventriculotomy was closed and the heart de-aired. Post-operative TOE confirmed absence of flow across the region of the VSD prior to closure of the sternotomy (Figure 5). Total CPB time was 45 minutes and cross clamp time 31 minutes. The patient was returned to the intensive care unit and made an uneventful recovery. He was discharged home on day 5.

Post-operatively, the patient’s breathlessness improved and he was able follow a full cardiac rehabilitation regimen. Follow-up TTE and cardiac CT were performed at 3 months to assess the repair. TTE highlighted a residual VSD within the apical septum, which was confirmed by cardiac CT to measure 20 mm × 7 mm, partly bounded by the moderator band. The previous repair was noted overlying the RV free wall. In light of these findings, which were out of keeping with the patient’s symptomatic improvement, a second opinion was sought at a quaternary centre. Now 9 months after the VSD repair, repeat TTE revealed normal LV size with good systolic function (EF 57%) despite a localised area of posterior wall akinesis secondary to the initial trauma. A muscular VSD was demonstrated in the mid apical septum with a maximum diameter of 13 mm, although there was no flow through the defect and it was almost completely closed on the side of the RV cavity by the pledgeted stitches of the previous surgical repair. Two small residual VSDs were noted along the approximation stitches with minimal left to right shunting. The peak gradient across the defect was 160 mmHg and there was no right-sided hypertension. Multidisciplinary assessment concluded no further closure was required owing to the presence of minimal residual shunt. The patient was managed conservatively with annual review and repeat transthoracic echo.

## Conclusions

This case highlights the complexity involved in both the diagnosis and management of traumatic ventricular septal defects (VSDs), particularly when located in the apical interventricular septum. It is well recognised that VSDs in this portion of the muscular septum may be difficult to address given extensive trabeculation within the RV [9], evidenced in this case by the presence of a prominent moderator band. The choice of the most appropriate exposure is made even more complex in cases of traumatic VSDs where the defect may follow an oblique path through the septum. Surgical approaches have included right ventriculotomy (longitudinal or transverse) [2], right atriotomy [10], left ventriculotomy [11], and right ventricular apical infundibulotomy [12]. Right atriotomy is the most common treatment of choice for perimembranous VSDs, whereby it avoids the risk of ventricular scarring and subsequent right bundle branch block (RBBB). However, this approach may not provide adequate exposure in muscular VSDs and may risk damage to the tricuspid valve chordae and leaflets. Whilst left ventriculotomy can provide good exposure, there is a risk of damage to major coronary vessels, long-term impairment of ventricular function, and postoperative arrhythmia secondary to ventricular scaring. More recently, Stellin et al. have proposed right ventricular apical infundibulotomy through a longitudinal incision to the infundibular apical free wall as an alternative approach to apical muscular VSDs [12]. This incision has the advantage of avoiding major conduction pathways and coronary vessels whilst also preserving RV mechanical function and providing excellent exposure [12]. In this case, we opted for a low right ventricular approach, not dissimilar to the apical infundibulotomy previously described, performed longitudinally adjacent to the distal LAD (Figure 1).

Once adequately exposed, the choice of repair technique is largely dependent on the location and size of the defect. Trans-cutaneous interventional closure (TCI) with devices such as the range of Amplatzer systems is widely utilized in the treatment of adult congenital VSDs with success in up to 97% of cases and low rates of major complications [13],[14]. However, there is less evidence surrounding their efficacy in traumatic VSD closure. A recent literature review showed that TCI for VSD closure was most successful in simple defects with a diameter less than 1.5 cm that underwent the procedure more than 4 weeks after the insult [6]. Conversely however, where the defect is in the perimembranous septum, follows an oblique course or is bounded by friable myocardium, TCI may be technically challenging, complicated by device embolisation, and associated with a high risk of post-procedural heart block [14]. In this case, although the defect was situated within the muscular septum, open surgical intervention was considered to be the most appropriate first line treatment. This was due to the presence of extensive trabecular tissue and the proximity of the moderator band, which provided a sub-optimal landing zone for device placement.

Surgical repair may be either by direct approximation or patch closure of the defect. Patch closure is conventionally performed with synthetic material such as Dacron or Gore-tex, which is readily available, and has the potential advantage of reducing the risk of residual VSD by stimulating fibrosis around the repair [15],[16]. Biological materials including glutaraldehyde treated autologous [16] and bovine pericardium [17] have also been less frequently used, however fresh pericardium is not recommended due to its potential to calcify, stretch and become aneurysmal over time. In this case, the location of the VSD just inferior to the moderator band provided minimal landing zone on which to secure a patch repair. As a result, and given the relatively small size of the defect, direct closure was performed with a pledgeted 4–0 proline suture.

Following repair, intraoperative TOE is advocated to confirm complete closure. However, although intraoperative TOE provides an invaluable real-time assessment of surgical repair in these cases, its results may be subjective, not entirely reproducible and operator dependent, particularly in less experienced centres [18]. In this case, TOE was unable to visualise complete closure of the defect, as, by definition, this was a partial thickness closure performed by direct approximation of the edges of the defect through the RV. Successful resolution of the VSD was therefore confirmed by demonstrating an absence of Doppler flow across the septum, without demonstration of any residual shunt.

This case highlights the importance of vigilant assessment and follow-up following cardiac trauma. Firstly, whilst the majority of clinically significant VSDs become evident in the first month after injury, not all traumatic VSDs are apparent on initial presentation to the emergency department; either due to delayed rupture of the intraventricular septum or, as in this case, due to the concomitant presence of life-threatening penetrating thoracic injury requiring emergent cardiopulmonary bypass [19],[20]. Furthermore, not all patients diagnosed with VSD will require interventional management in the first instance. Indeed, conservative management is advocated in asymptomatic muscular VSDs where, in the absence of pulmonary hypertension, ventricular dimensions remain normal and Qp:Qs remains <2. Furthermore, small VSDs in young, otherwise healthy individuals may close spontaneously over time [21]. As such, these patients may be managed remotely with regular symptomatic review and echocardiographic assessment. Conversely however, spontaneous closure is unlikely in older patients, or in larger VSDs where there is a significant shunt. In these cases early intervention is recommended to prevent disease and symptom progression.

Regardless of the management strategy, repeated re-assessment and evaluation is vitally important. Following surgical or device intervention, follow-up echocardiography is fundamental to ensure adequate closure and the absence of residual VSD. In our practice, after surgical VSD repair trans-thoracic echo (TTE) is performed prior to discharge. This is then repeated at 3 months follow-up when the patient returns to clinic for full clinical assessment, provided the initial recovery is uneventful. Beyond this, we advocate yearly echocardiographic and clinical follow-up if any residual defect is present. In the absence of residual defect, follow-up echocardiography is performed on the basis of the patient’s clinical status and is advocated primarily, but not exclusively, where there is increasing breathlessness, worsening exercise tolerance, or novel auscultatory findings. In addition, in symptomatic cases without any echocardiographic evidence of residual defect, adjunctive measures such as the 6-minute walk test can be used to provide a functional assessment where more detailed anatomical investigation is likely to be warranted [22].

In summary, this case highlights the potential pitfalls in both the diagnosis and management of traumatic VSDs particularly where the patient presents *in extremis* with other life-threatening injuries. Furthermore, it exemplifies the importance of a multidisciplinary approach when planning any elective intervention. Subsequent to surgical intervention, repeated re-assessment and re-evaluation is vital in these patients, and it is of paramount importance to ensure vigilant long-term follow-up is provided.

## Consent

Written informed consent was obtained from the patient for publication of this case report and any accompanying images. A copy of the written consent is available for review by the Editor-in-Chief of this journal.

## Authors’ original submitted files for images

Below are the links to the authors’ original submitted files for images. Authors’ original file for figure 1 Authors’ original file for figure 2 Authors’ original file for figure 3 Authors’ original file for figure 4 Authors’ original file for figure 5

## Abbreviations

VSD: Ventricular septal defect
TTE: Trans-thoracic echocardiography
TOE: Trans-oesophageal echocardiography
RV: Right ventricle
LV: Left ventricle
RA: Right atrium
LAD: Left anterior descending artery
TCI: Trans-cutaneous intervention (closure)
EF: Ejection fraction
CT: Computerised tomography
CPB: Cardio-pumlonary bypass
PVel: Peak velocity
PGrad: Peak gradient
RBBB: Right bundle branch block
MCA: Middle cerebral artery
Qp: Qs: Pulmonary flow: Systemic flow
