# Supplementary figures and images for: Late surgical repair of a traumatic ventricular septal defect
Source: J Cardiothorac Surg. 2014 Sep 20;9:145. doi: 10.1186/s13019-014-0145-1 (PMC4198620; doi:10.1186/s13019-014-0145-1)

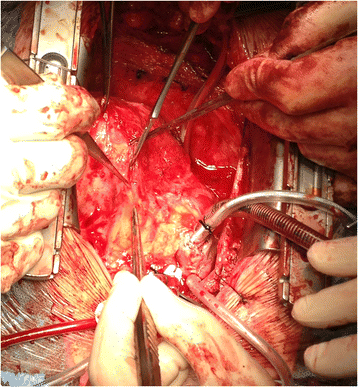

Supplement: Supplementary file 1 — Authors’ original file for figure 1 [file 13019_2014_145_MOESM1_ESM.gif]

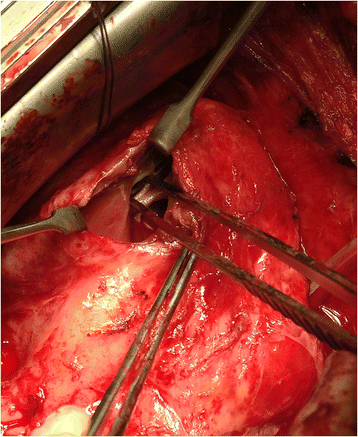

Supplement: Supplementary file 2 — Authors’ original file for figure 2 [file 13019_2014_145_MOESM2_ESM.gif]

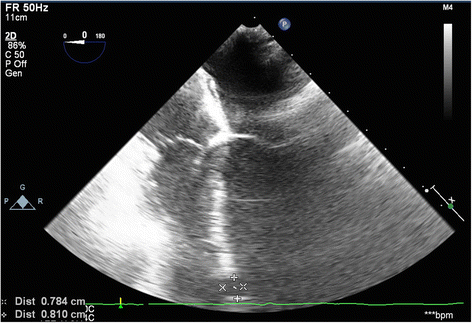

Supplement: Supplementary file 3 — Authors’ original file for figure 3 [file 13019_2014_145_MOESM3_ESM.gif]

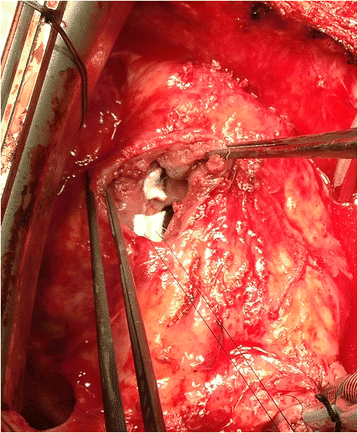

Supplement: Supplementary file 4 — Authors’ original file for figure 4 [file 13019_2014_145_MOESM4_ESM.gif]

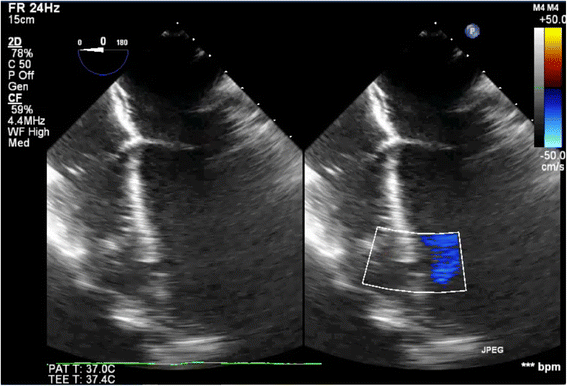

Supplement: Supplementary file 5 — Authors’ original file for figure 5 [file 13019_2014_145_MOESM5_ESM.gif]
